# Can carbon emissions trading policy promote product bargaining power increases for high-carbon enterprises? Evidence from China

**DOI:** 10.1371/journal.pone.0302916

**Published:** 2024-06-17

**Authors:** Sen Wang, Jinye Li

**Affiliations:** 1 School of Economics and Management, Xinjiang University, Urumqi, China; 2 Research Center for Macroeconomic High-Quality Development of Xinjiang, Urumqi, China; Krirk University, THAILAND

## Abstract

Carbon emissions trading policies play a crucial role in facilitating the transition to high-end products within high-carbon enterprises. Nevertheless, current empirical analyses of the carbon emissions trading market exhibit a lack of precision and are susceptible to bias in their findings. Limited research has been conducted on the influence of product quality as a potential constraint on the impact of carbon trading on product bargaining power. This study presents a double-difference model utilizing data on emission-control enterprises in China’s carbon market to examine the influence of the carbon emissions trading mechanism on the bargaining power of high-carbon products. Empirical analysis is conducted using financial data from listed companies in China spanning the years 2010 to 2020. The findings indicate that the implementation of carbon emissions trading policies has a dampening impact on the product bargaining power of high-carbon enterprises. Moreover, carbon emissions trading policies have heterogeneous effects on the product bargaining power of high-carbon firms with different life cycles, with mature high-carbon firms receiving a boost and declining high-carbon firms receiving a dampening effect. Mechanism test finds that the incomplete transmission effect of cost shocks resulting from carbon emissions trading policies has negatively affect the product bargaining power of high-carbon enterprises. Further research finds that product quality is a key factor in determining the effect of the carbon emissions trading policy, and that the impact of the carbon emissions trading policy on the bargaining power of products of high-carbon firms takes on a "U" shape due to product quality. Once the product quality exceeds the bottleneck value of 0.5956, the policy significantly increases the bargaining power of products. The study confirms that the establishment of carbon markets can effectively increase the bargaining power of superior products. These results offer a comprehensive theoretical and practical foundation for nations to advance the development of carbon markets and facilitate the achievement of sustainable development by high-carbon enterprises.

## 1. Introduction

One of the primary global challenges confronting contemporary society is the imperative of attaining sustainable and resilient development amidst the backdrop of climate change. This necessitates a concerted effort by nations to intensify measures aimed at curbing carbon emissions in order to effectively address this pressing global issue [1, 2]. China, being one of the foremost contributors to carbon dioxide emissions on a global scale, bears a significant obligation to mitigate its emissions output [3, 4]. At the 75th session of the United Nations General Assembly, China articulated its "30–60" dual-carbon target, which aims to reach a peak in carbon emissions by the year 2030 and achieve complete carbon neutrality by 2060. In order to advance the objectives of environmental sustainability and reduced carbon emissions, the Chinese government incorporated the establishment of a unified national carbon market into the government work report of 2021, thereby elevating efforts towards carbon emission reduction to an unprecedented degree. Nevertheless, China confronts obstacles in enhancing energy efficiency and curbing carbon emissions, notably including a subpar level of technological advancement [5]. Presently, China’s energy use efficiency stands at approximately 30% of that of developed nations, with its total energy consumption consistently ranking as the highest globally [6]. China continues to manufacture numerous energy-intensive, low-end products that lack export competitiveness and contribute to excessive energy consumption, resulting in significant negative environmental impacts. Consequently, there is an urgent need for the high-end transformation of China’s high-carbon products [7].

The high-carbon sector, as a major contributor to greenhouse gas emissions, plays a crucial role in reducing carbon emissions and advancing sustainable development [8]. The carbon emissions trading policy functions as a market mechanism that offers incentives for the substantial transformation of high-carbon industries [9]. Both the Chinese and European Union carbon markets share similar foundational principles, including the establishment of a cap on total carbon emissions, the implementation of carbon quota control through market trading, and the selection of emission-control enterprises for participation based on historical emission intensity. The implementation of the carbon emissions trading policy, regulation of high energy-consuming enterprises, promotion of low-carbon technological innovation, and encouragement of enterprise product structure upgrades provide a new impetus and direction for the high-carbon industry, guiding high-carbon enterprises towards producing more environmentally friendly and higher-end products [10, 11]. The high-carbon industry, a significant component of the economy, is currently confronted with dual challenges stemming from environmental regulations and global competition. Consequently, addressing the imperative of leveraging the market incentive advantages inherent in carbon emissions trading policies to steer high-carbon enterprises towards producing superior quality goods and bolstering their pricing capabilities has emerged as a pressing issue requiring immediate resolution [12].

The concept of product bargaining power is considered a crucial factor in determining export competitiveness within high-carbon industries. Effective product bargaining power enables enterprises to secure more favorable pricing positions in the global market, thereby strengthening their overall export competitiveness [13]. In order to achieve a harmonious equilibrium between carbon emission regulation and product bargaining power, as well as to comprehend the pivotal role in fostering synergistic growth of environmental and economic advantages, it is imperative to undertake a comprehensive examination of the correlation between carbon emission trading policies and product bargaining power within the high-carbon sector, and to devise appropriate policies and strategies accordingly.

Current research primarily examines the influence of carbon emissions trading policies on the product bargaining power of high-carbon enterprises through the lens of environmental cost shock transfer. Baccianti and Schenker’s study on EU countries reveals that carbon regulatory measures typically diminish the bargaining power of exporting firms, leading to a decrease in profit margins as firms struggle to offset the impact of cost compensation [14]. However, Marin et al. [15] posit a contrasting perspective in their examination of high-carbon enterprises in Europe, contending that carbon emissions trading policies can potentially offset costs and enhance product bargaining power through the innovation compensation effect. Given the rapid pace of innovation and technology transfer limitations in developed nations, Chinese enterprises are increasingly prioritizing the optimization of production costs as a crucial strategy for enhancing market competitiveness, ultimately leading to market success and increased negotiating leverage [16, 17].

In conclusion, while some studies have undertaken initial inquiries into the effects of carbon emissions trading policies on product bargaining power, three research deficiencies remain to be rectified. Firstly, existing studies have failed to designate emission-control enterprises in the carbon emissions trading market as the experimental group for analysis, instead opting to include all enterprises from pilot provinces, cities, and other regional levels. The efficacy of China’s carbon emissions trading policy in enhancing the product bargaining power of high-carbon enterprises remains inconclusive within the context of varying international policies. Further research is needed to ascertain the impact of product quality on the bargaining power of enterprises’ products, as existing studies have not adequately addressed this aspect, highlighting a notable research gap in this area. Compared with the existing research, three new contributions were made to fill current research gaps as follows. This paper adopts a research perspective focused on the reduction of carbon emissions and the increase in prices. It examines the direct effects of carbon emissions trading policies on product bargaining power through both theoretical analysis and empirical evidence. Additionally, the study explores the role of product quality as a limiting factor in the influence of carbon emissions trading policies on product bargaining power. The aim is to address the emerging question of how carbon emissions trading policies impact the product bargaining power of high-carbon enterprises. Furthermore, this study employs a difference-in-difference model (DID) to analyze the impact of the timing of high-carbon enterprises entering the list of emission-control enterprises in the carbon market on their product bargaining power. Utilizing data from listed companies spanning from 2010 to 2020, we aim to investigate the micro-level effects of the carbon trading mechanism, thereby enhancing the depth of our research. Furthermore, the research delves into the impact of heterogeneity on the export high-carbon industry by examining the life cycle of an enterprise and industry factors. This analysis offers a comprehensive theoretical and practical foundation for facilitating the high-end transformation of high-carbon enterprises and bolstering the competitiveness of the high-carbon industry on a global scale.

## 2. Literature review and theoretical hypotheses

### 2.1 The direct impact of CETP on H-C enterprises PBP

The cost compliance hypothesis within neoclassical economic theory posits that environmental regulatory policies will elevate private production expenses and diminish enterprise competitiveness [18]. Furthermore, the bargaining power of high-carbon enterprises is influenced by the cost-compliance impact of carbon emission regulations. More specifically, carbon emissions trading policies introduce a novel cost restriction to firm profit optimization [19]. Carbon emissions trading policies introduce a cost constraint that impacts firms’ profit maximization [20]. The imposition of carbon emission quota constraints can effectively regulate and limit production operations and energy consumption behaviors of enterprises, leading to adjustments in the production practices of high-carbon firms. This adjustment incurs increased production costs, ultimately diminishing the firms’ ability to negotiate product prices [21]. Based on Porter’s hypothesis, the implementation of effective carbon emission regulations can incentivize businesses to innovate in order to enhance production efficiency, thereby mitigating the financial burden associated with such regulations, known as the innovation compensation effect [17]. Carbon emission quota constraints have a positive effect on enterprises to increase R&D investment, but the compensation of production efficiency brought by innovation has a time lag, and the large amount of capital required for technological innovation and production process upgrading may have a crowding-out effect on the current factor inputs, weakening the production efficiency, and then negatively affecting the bargaining power of products [22]. Furthermore, the inability of enterprises to successfully leverage technological innovation for the purpose of gaining competitive advantages in their products may result in a diminished bargaining power during price negotiations [23]. Additionally, viewing carbon emissions trading policies as a form of market-based environmental regulation, enterprises have the opportunity to alleviate the strain of carbon quotas through participation in carbon markets. However, this may also lead to a slowdown in production adjustments and pose challenges in mitigating the adverse effects on product bargaining power.

In conclusion, the impact of environmental regulations on firm competitiveness is multifaceted. The existing literature on the strong Porter’s hypothesis is contentious, with greater recognition of its effects. This study posits that the "weak Porter’s hypothesis" effect of the carbon emissions trading mechanism is indeed beneficial, yet the imposition of carbon quotas may elevate production adjustment costs for enterprises, consequently diminishing product bargaining power. Based on this, the article proposes hypothesis 1: carbon emissions trading policies may result in a notable reduction in the market influence of high-carbon products.

### 2.2 Mechanisms for impact of CETP on H-C enterprises PBP

The sticky price theory posits that prices of goods and services in markets exhibit slow and lagged adjustments relative to costs [24]. Fluctuations in firms’ production costs may arise from production adjustment costs resulting from the carbon trading mechanism and increased R&D inputs. The menu cost effect suggests that cost shocks experienced by high-carbon firms are not fully passed on to product prices, leading to lower price volatility compared to cost volatility [25]. Moreover, high-carbon enterprises may strategically choose not to fully transfer the increased production costs resulting from carbon emissions trading policies to consumers in order to maintain their export market share amidst market competition and other influences [22]. Consequently, the impact of these policies on product prices may be mitigated, leading to a disparity between cost and price increases and potentially diminishing the enterprises’ ability to negotiate prices.

In conclusion, a thorough analysis of the factors influencing product bargaining power is conducted by integrating the carbon emissions trading mechanism. It is determined that the production cost and product price of enterprises serve as significant influencing mechanisms of carbon quota constraints on product bargaining power. Furthermore, the heterogeneous impact of the carbon emissions trading mechanism on the production cost and product price of enterprises is identified as crucial in the reduction of product bargaining power. Building upon this analysis, the article posits hypothesis 2: Carbon emissions trading policies may intensify the degree of this decline.

**Fig 1:**
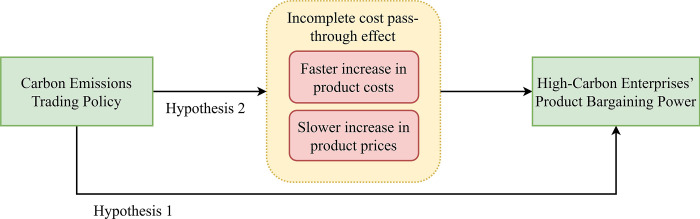
A schematic representation illustrating the impact of carbon emissions trading policies on the product bargaining power of high-carbon firms.

In conclusion, Fig 1 illustrates the diagram outlining the mechanisms through which carbon emissions trading policies impact the product bargaining power of high-carbon firms.

### 2.3 Product quality bottleneck effect of CETP on H-C enterprises PBP

Sellers possess superior knowledge regarding goods compared to buyers in product markets, allowing them to capitalize on the market by providing trustworthy information to buyers. Utilizing product quality as a market signal can partially address the issue of information asymmetry [26]. Enhancing product quality enables firms to mitigate transaction uncertainty stemming from information asymmetry, thereby influencing the firm’s bargaining power within the market. The theory of endogenous decision-making quality posits that enhancements in product quality may yield varying effects on both product prices and production costs, thereby influencing the pricing capabilities of high-carbon enterprises within the context of carbon emissions trading policies [27]. This perspective underscores the relationship between product quality and pricing strategies employed by high-carbon enterprises. When a certain level of product quality is achieved, the enterprise gains a competitive advantage in terms of quality over similar products, leading to an increase in pricing elasticity for the enterprise’s product. Consequently, improvements in product quality can result in price spillover. The flexible adjustment of product prices can partially offset the costs associated with carbon emissions trading policies, thereby mitigating the negative impact on product pricing ability and enabling enterprises to gain a competitive edge in the market [28, 29]. From the perspective of how product quality impacts the production costs of high-carbon enterprises, it is observed that enhancing product quality typically results in higher production costs. This increase may diminish the cost competitiveness of enterprises offering similar products and potentially amplify the financial implications of carbon emissions trading policies [30]. Therefore, it is evident that there exists a distinction in the influence of "low-end improvement" and "high-end improvement" of product quality on the pricing power of high-carbon enterprises, indicating that the pricing power of products can experience significant enhancement only when product quality reaches a high standard [26, 31]. Consequently, the moderating impact of product quality may exhibit non-linear characteristics.

The article proposes hypothesis 3, suggesting that product quality may exhibit an "inverted U" bottleneck effect that is both intensified and mitigated by the impact of carbon emissions trading policies on the pricing power of high-carbon enterprises.

**Fig 2:**
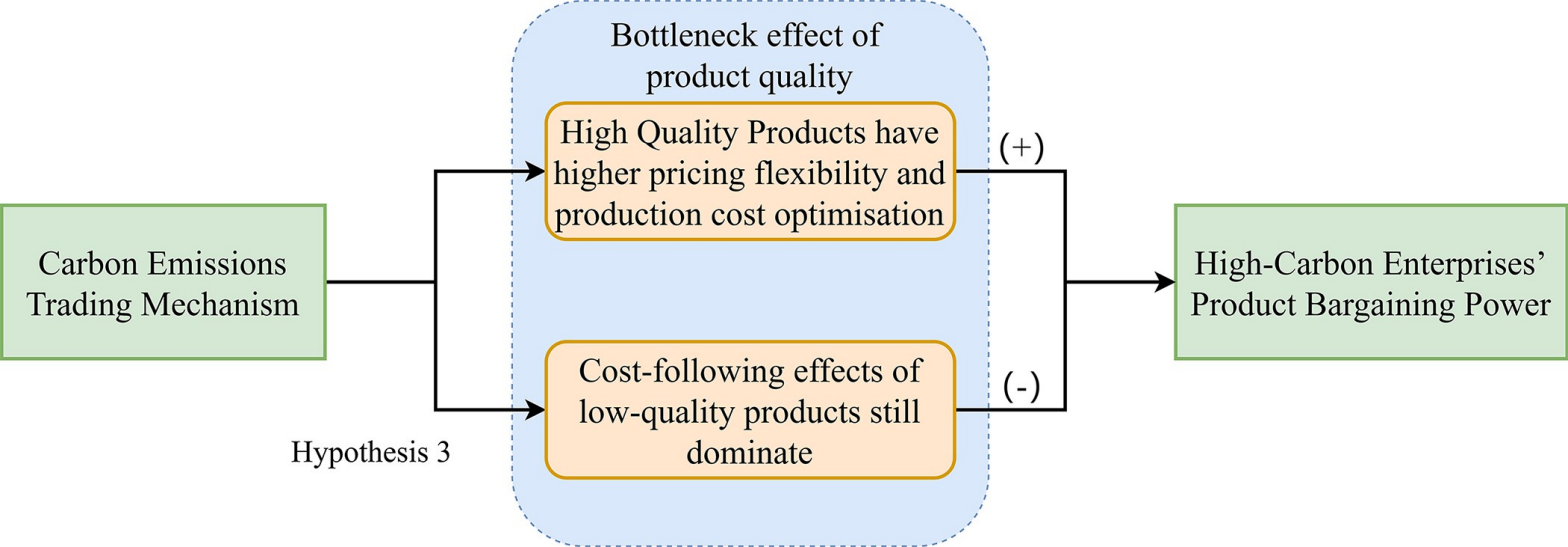
The mechanism diagram illustrating the impact of carbon trading policies on the product bargaining power of high-carbon firms through the bottleneck effect on product quality.

To summarize, Fig 2 illustrates the impact of the bottleneck effect on product quality within carbon emissions trading policies, specifically on the pricing capabilities of high-carbon enterprises.

## 3. Model setting and data sources

### 3.1 Sample and data

Since 2013, the Chinese government has incrementally introduced carbon market trading in several regions, including Shanghai, Beijing, Guangdong, Hubei, Tianjin, Chongqing, and Fujian, culminating in the establishment of a national carbon market for the power generation sector in 2021. Given the similarity in the implementation mechanism of the current cap-and-trade policy to that of the pilot period, this study primarily examines the carbon market in these seven regions. This study utilizes data from high-carbon listed companies in the Shanghai and Shenzhen A-shares markets as the primary sample, selecting the time frame of 2010–2020 to allow for a minimum two-year period prior to the establishment of each carbon market. The data analyzed in this research primarily consists of the inclusion of high-carbon listed companies in carbon market emission control entities and enterprise-level data, with the former being manually compiled based on carbon market information and the latter being sourced from the China Stock Market & Accounting Research Database (CSMAR) matching data.

The initial samples are screened based on the principles of (1) excluding samples with information loss and (2) retaining samples from high-carbon listed enterprises. In order to mitigate the impact of extreme data values on estimation results, this study applies a shrinkage treatment to all continuous variables at the 1% and 99% quantiles.

### 3.2 Model selection

#### 3.2.1 Benchmark model

In this study, the export products of listed enterprises within the emission-control enterprises list in China’s carbon market pilot are designated as the treatment group, while the export products of other listed enterprises are assigned as the control group. Subsequently, a Difference-in-Differences (DID) model is developed to conduct an empirical analysis based on the timing of each listed enterprise’s inclusion in the emission-control enterprises list within the carbon trading market. The data on enterprise destinations from 2010–2020 is utilized to analyze the influence of the policy on the emissions control enterprises’ products in the pilot program. Subsequently, a Differences-in-Differences (DID) model is developed to assess the specific impact of the carbon emissions trading policy on the bargaining power of high-carbon enterprises:

$$mkp_{it}=\varphi_{0}+\varphi_{1}did_{it}+\varphi_{2}x_{it}+\mu_{i}+\delta_{t}+\varepsilon_{it}$$ (1)

The dependent variables *mkp*_*it*_ in model (1) represent the product bargaining power of enterprise product *i* in year *t*. *did*_*it*_ is *treat*_it*_post_*it*_, which represents the direct effect of the policy, *treat*_*it*_ is a province dummy variable; *post*_*it*_ is a year dummy variable. *x*_*it*_ is a control variable, *μ_it_* and *δ_it_* control for enterprise fixed effects and time-fixed effects, respectively, *ε*_*it*_ is a random disturbance term, and robust standard errors are clustered at the enterprise level.

#### 3.2.2 The model of incomplete cost pass-through effects

This paper aims to assess the incomplete transmission effect of carbon emissions trading policy on the bargaining power of products by utilizing a model (1) that replaces explanatory variables with product price or production cost to construct and test model (2):

$$PP_{it}|PC_{it}=\alpha_{0}+\alpha_{1}did_{it}+\alpha_{2}x_{it}+\mu_{i}+\delta_{t}+\varepsilon_{it}$$ (2)

In model (2), mechanism variables *PP_it_* or *PC_it_* are product unit price or product marginal cost, and the coefficient *α*_1_ is used to indicate the imbalance change of product cost and product price brought about by the carbon emissions trading policy as a measure of the cost imperfect transmission effect caused by the implementation of the policy, and the other variables have the same meanings as in model (1).

#### 3.2.3 Product quality bottleneck effect model

This study expands upon the concept of product quality bottlenecks by investigating the influence of carbon emissions trading policies on product markup rates across varying product quality conditions.

To achieve this, the study incorporates product quality primary and square terms into a model (1) and develops the DDD model (model 3) to assess the nonlinear moderating effect of product quality on the relationship between carbon emissions trading policies and product markup rates. Additionally, in the presence of a nonlinear moderating effect of product quality, group dummy variables should be established based on the inflection point value of the product quality moderating effect. Subsequently, the bottleneck effect of product quality can be examined by incorporating these dummy variables into model (1). Specifically, a product quality grouping dummy variable (*quall* or *qualh*) is introduced in conjunction with the policy dummy variable *did* to assess the influence of carbon emissions trading policy on product markup rates across varying levels of product quality. Among them, *quall* denotes the low product quality group, when qua is smaller than the inflection point value of 1, and larger than the inflection point value of 0. *qualh* is the high product quality group when qua is larger than the inflection point value of 1, and smaller than the inflection point value of 0.

The form of model (3) is set as follows:

$$mkp_{it}=\beta_{0}+\beta_{1}did_{it}+\beta_{2}qua_{it}+\beta_{3}did_{it}*qua_{it}+\beta_{4}qua_{it}^{2}+\beta_{5}(did_{it}*qua_{it}^{2})+\beta_{6}x_{it}+\mu_{i}+\delta_{t}+\varepsilon_{it}$$ (3)

Eq (3), $qua_{it}^{2}$ is the squared term for the level of product quality; the interaction terms $did_{it}*qua_{it}$ and $did_{it}*qua_{it}^{2}$ represent the moderating effect of product quality on the direct effect of the policy, and the other symbols have the same meaning as in Eq (1).

### 3.3 Variable selection

#### 3.3.1 Product bargaining power (*mkp*)

In this paper, we measure high-carbon enterprises’ product bargaining power in terms of firm-level cost markups using De Lecker and Warzynski’s methodology [32–34], which first calculates the variable input-output elasticity *θ_iqt_* of firm q in city i in year t by industry classification of the high-carbon sector, and then divides the variable input-output elasticity *θ_iqt_* by the share of expenditures *α_iqt_* to obtain the firm markups rate *μ_iqt_* of firm q in city i in year t.


$$mkp_{iqt}=\mu_{iqt}=\theta_{iqt}/\alpha_{iqt}$$ (4)

#### 3.3.2 Carbon emissions trading policy (*did*)

The explanatory variable *did*_*it*_ is the interaction term between *treat*_*it*_ and *post*_*it*_, which represents the carbon emissions trading policy dummy variable, where *treat*_*it*_ indicates whether the listed company is a controlled-emission enterprise in the carbon market [35]. We manually collect and organize the emission control enterprises of each carbon market disclosure data, and then match them with the listed companies, if the listed company is an emission control enterprise, it is defined as the treatment group and assigned a value of 1 to *treat*_*it*_, and the others are categorized into the control group, and assigned a value of 0 to *treat*_*it*_. Variable *post*_*it*_ is a dummy variable for the time when the enterprise entered the carbon market control list, with the year after the enterprise was included in the carbon market control list assigned a value of 1, and the year before the inclusion of the list and other enterprises that did not enter the control list assigned a value of 0 [36, 37].

#### 3.3.3 Control variables (*CV*)

To analyze the impact of carbon trading market policies more fully on product bargaining power, it is also necessary to set control variables that may have an impact: (1) Enterprise capital intensity (capital), measured using the ratio of net fixed assets to the number of employees [38]. (2) Enterprise wage per capita (wage), measured using the ratio of enterprise employee compensation to the number of employees [39]. (3) Enterprise total factor productivity (tfp) measured using the LP and ACF methods to estimate the total factor productivity of the enterprise [40]. (4) Enterprise size (size), based on the total assets of enterprises measured by taking the logarithms [41]. (5) Enterprise ownership (soe), defined as state-owned and non-state-owned enterprises based on information from the database of listed enterprises [42]. (6) Industry concentration (HHI) measured using the Herfindahl index at the industry level [43]. Descriptive statistics for the main variables are shown in Table 1.

**Table 1:** Descriptive statistics.

*Variable*	*Variable meanings*	*Obs*	*Mean*	*Std*.*Dev*.	*Min*	*Max*
*mkp*	High-carbon enterprises’ product bargaining power	5280	2.3886	0.7592	1.0297	4.9985
*did*	Carbon emissions trading policy	5280	0.0615	0.2148	0	1
*capital*	High-carbon enterprise capital intensity	5280	2.2348	1.7993	0.1163	27.9946
*wage*	High-carbon enterprise wage per capita	5280	9.3357	1.1222	3.1082	15.3960
*tfp*	High-carbon enterprise total factor productivity	5280	5.9812	0.9379	0.9478	12.7017
*size*	High-carbon enterprise size	5280	22.255	1.4296	17.2770	26.8060
*soe*	High-carbon enterprise ownership	5280	0.4862	0.4999	0	1
*hhi*	Industry concentration	5280	0.1498	0.1028	0.0443	0.8435

## 4. Empirical findings and analysis

### 4.1 Baseline regression

The analysis in Table 2 reveals the significant negative impact of carbon emissions trading policy on the bargaining power of high-carbon enterprises, as indicated by the coefficient of -0.0675 in column (2) of the regression results, which is statistically significant at the 5% level. The findings suggest that the policy leads to an increase in production costs for high-carbon enterprises due to environmental cost shocks, as well as an increase in research and development costs through the promotion of innovation within these enterprises. However, the inability to fully transfer these cost shocks to product prices results in the predominance of the cost-following effect, ultimately diminishing the bargaining power of high-carbon products [44]. The results support the validity of hypothesis 1.

**Table 2:** Direct effects of carbon emissions trading policies.

*variables*	(2)
	*mkp*
*did*	-0.0675**
	(0.0310)
*capital*	-0.0007
	(0.0008)
*wage*	-0.0779**
	(0.0305)
*tfp*	0.2373***
	(0.0315)
*size*	1.0846***
	(0.1160)
*soe*	0.1521*
	(0.0897)
*hhi*	0.0825
	(0.1876)
*Enterprise FE*	*Yes*
*Time FE*	*Yes*
*Obs*.	5280
*R* ^*2*^	0.8054

Note: *, **, *** denote significance levels of 10%, 5%, and 1% respectively, and values in parentheses denote robust standard errors.

The carbon emissions trading policy significantly influences the production of high-carbon enterprises through two primary mechanisms: the cost-following and innovation compensation effects of carbon emission quota constraints, and the market allocation effect of carbon emissions trading. This policy leverages market mechanisms to reduce the marginal cost of production adjustments and emissions reductions for high-carbon enterprises, thereby achieving industry-wide carbon emission control in the present year. Seller firms achieve greater economic benefits through the implementation of cleaner production practices, while buyer firms meet carbon emission reduction mandates by procuring carbon emission allowances, leading to a mutually advantageous outcome [45]. Our findings indicate that the carbon emissions trading policy diminishes the bargaining power of high-carbon firms in the marketplace. The policy’s impact on the product bargaining power of high-carbon enterprises may be attributed to the lack of a clear innovation compensation effect during the initial stages of production adjustment. However, research suggests that the carbon emissions trading policy, as a market-oriented environmental regulation, can mitigate the cost-following effect in the early stages of production adjustment. Further investigation is needed to identify additional factors contributing to the policy’s impact on high-carbon enterprises’ product bargaining power [46].

### 4.2 Robustness tests

#### 4.2.1 Parallel trend test

**Fig 3:**
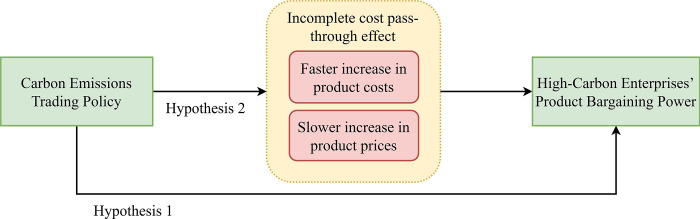
Parallel trend test of product bargaining power.

The foundational premise of the difference model necessitates the absence of a substantial disparity between the experimental and control groups prior to the implementation of the policy. In order to investigate this, the study references Beck et al. [47], designating period 1 as the baseline period before the introduction of the entry list policy, and aggregating data for firms up to 5 years post-inclusion in the carbon market list into period -5, and data for firms 3 years post-inclusion into the carbon market list into period 3. The analysis presented in Fig 3 indicates that there is no statistically significant difference in trends between the two sample types prior to and following the inclusion of high-carbon firms in the roster of emission-control firms. Furthermore, the significance level of the bargaining power of products from high-carbon firms experiences a notable increase upon their inclusion in the list of firms.

#### 4.2.2 Placebo test with regression to baseline

**Fig 4:**
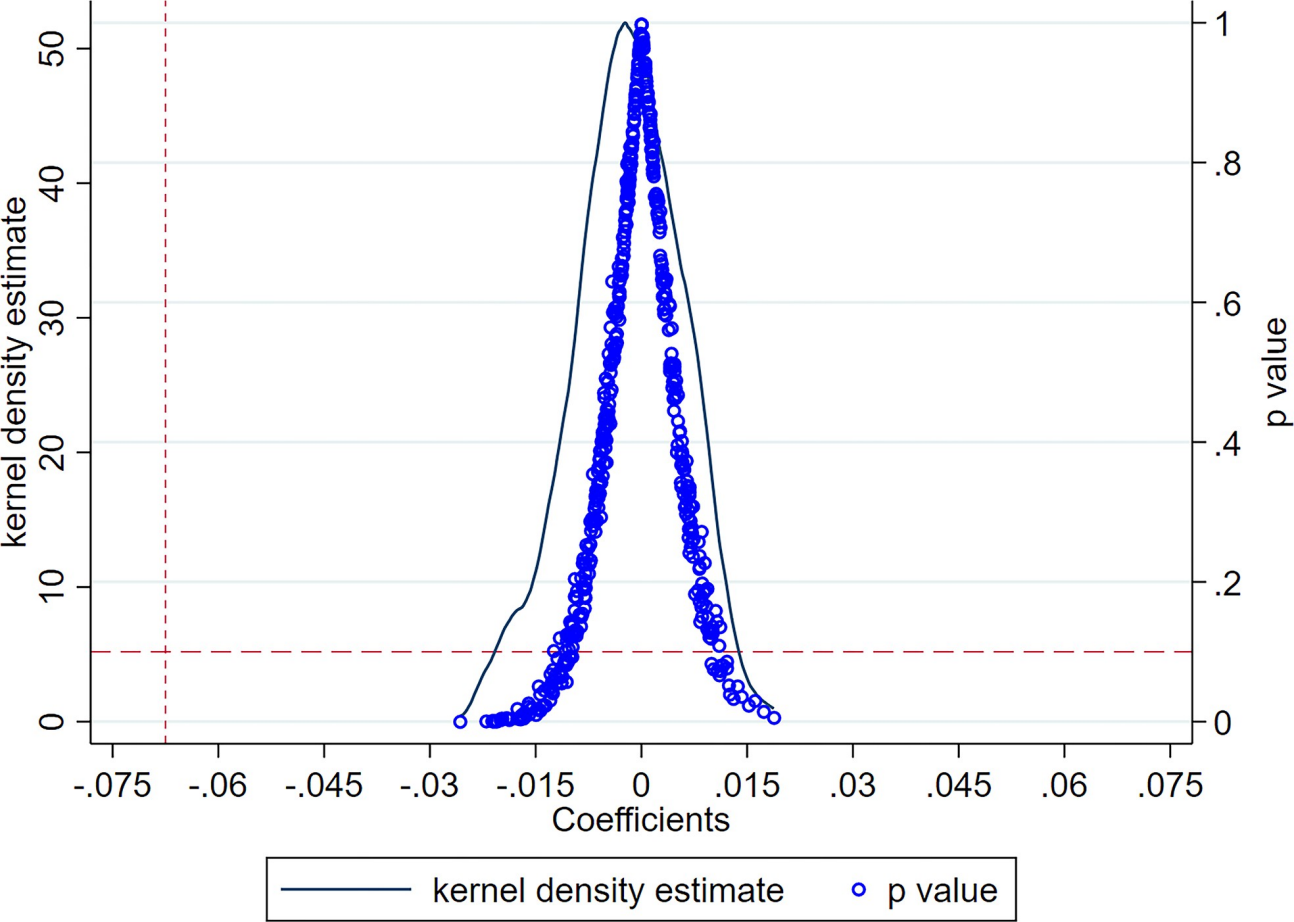
Placebo test with regression to baseline.

In order to assess the reliability of the Difference-in-Differences (DID) regression findings, a placebo test involving the creation of a simulated treatment group was conducted. The DID regression coefficients derived from this placebo test are presented in Fig 4. The findings reveal that all coefficient estimates are closely centered around zero, suggesting that extraneous variables do not significantly impact the baseline regression analysis examining the influence of carbon trading policies on the market power of high-carbon firms. This further underscores the robustness of the estimation results presented in the preceding section.

#### 4.2.3 Heterogeneity treatment effect test

The potential for heterogeneity bias in multiperiod Difference-in-Differences (DID) estimates is attributed to the double-differential estimation method used to compare post-treatment group with first-treatment group samples. In contrast, DID estimates derived from comparisons between the first-treatment group and the never-treated group, the post-treatment group and the never-treated group, and the first-treatment group and the post-treatment group do not result in estimation bias when the parallel-trend assumption is met [48]. In the context of the two-way fixed-effects framework, the credibility of multi-period Difference-in-Differences (DID) estimates remains intact even when estimates derived from samples of the post-treated and first-treated groups carry minimal weight. To assess the robustness of the benchmark regression results, this study employs the Bacon decomposition method and presents the findings in Table 3. According to the findings presented in Table 3, the weight assigned to the DID estimator for the "post-treatment group and pre-treatment group" is a mere 0.15%. This suggests that the inclusion of a two-way fixed-effects framework is unlikely to be significantly influenced by the presence of heterogeneous treatment effects, and the outcomes of the benchmark regression exhibit a degree of robustness.

**Table 3:** Results of the Bacon decomposition of the benchmark regression.

Grouping type of DID	*mkp*	
	DID estimator	weights
Treated group vs. untreated group	-0.0923	0.9965
Pre-treatment group vs. post-treatment group	-0.0521	0.0020
Post-treatment group vs. pre-treatment group	0.0376	0.0015

#### 4.2.4 Excluding other environmental policy shocks

After controlling for time-fixed effects and accounting for potential interference from other policy shocks in the same period, the article examines the impacts of the pilot low-carbon city policy, the implementation program of energy-saving and low-carbon actions of 10,000 enterprises, and the pilot policy of sewage trading on carbon emissions trading mechanism. The regression results in Table 4 indicate that the effects of the carbon emissions trading mechanism on product quality and bargaining power remain robust.

**Table 4:** Excluding other environmental policy shocks.

*variables*	(1)	(2)	(3)
	*mkp*	*mkp*	*mkp*
*did*	-0.0697**	-0.0656**	-0.0648**
	(0.0309)	(0.0308)	(0.0308)
*LCP*	*Yes*	*No*	*No*
*WCA*	*No*	*Yes*	*No*
*PTP*	*No*	*No*	*Yes*
*CV*	*Yes*	*Yes*	*Yes*
*Enterprise FE*	*Yes*	*Yes*	*Yes*
*Time FE*	*Yes*	*Yes*	*Yes*
*N*	5280	5280	5280
*R* ^*2*^	0.8054	0.7978	0.8049

#### 4.2.5 Changing the data time window period

Given that firms entering the control ranking in the year the carbon market opens may have their production decisions influenced by various factors both before and after entering the market, the data from the year the carbon emissions trading market opens is excluded from the sample to mitigate the impact of unobserved shocks. The remaining sample is then utilized in the regression analysis. The regression analysis presented in Table 5 indicates that the effects of the carbon emissions trading mechanism on the product bargaining power of high-carbon firms remain consistent with those observed in the benchmark regression, irrespective of the inclusion of control variables.

**Table 5:** Changing the data time window period.

*variables*	(1)	(2)
	*mkp*	*mkp*
*did*	-0.0809***	-0.0553*
	(0.0313)	(0.0315)
*CV*	*No*	*Yes*
*Enterprise FE*	*Yes*	*Yes*
*Time FE*	*Yes*	*Yes*
*N*	3856	3856
*R* ^*2*^	0.7978	0.7923

#### 4.2.6 Heterogeneity analysis

Subsequently, we investigate the impact of the carbon emissions trading policy on the product bargaining power of high-carbon firms through a heterogeneity analysis, considering the firm life cycle. By assessing key metrics such as sales revenue growth rate, capital expenditure rate, retained earnings rate, and firm age, we assign a total score to each firm and categorize them into growth, maturity, or decline stages [49]. The findings suggest that high carbon firms in the top 1/3 of scores are in the growth stage, those in the bottom 1/3 are in the decline stage, and those in the middle portion are in the maturity stage.

The regression analysis in columns (1)-(3) of Table 6 indicates a significantly positive coefficient of "did" for high-carbon firms in the maturity period, while a significantly negative coefficient is observed only for high-carbon firms in the decline period. This suggests that the influence of carbon emissions trading policies on the product bargaining power of high-carbon enterprises is closely tied to the stage of the enterprise’s life cycle. This relationship may be attributed to the fact that during the growth phase, high-carbon enterprises experience an increase in market demand, allowing them to rapidly acquire more customers and expand their market share. In response to the environmental pressures imposed by the carbon emissions trading policy, high-carbon enterprises are inclined to mitigate environmental costs by reducing production expenses and raising the markup on their products. During the mature stage of high-carbon firms, intense market competition necessitates the maintenance of existing market share and the pursuit of new growth opportunities. Carbon emission regulation serves to underscore the significance of product quality for both firms and consumers, prompting firms to align production practices with a quality-oriented approach and enhance the efficiency and cost-effectiveness of high-carbon products. In times of recession, market saturation, heightened competition, and diminished consumer demand may contribute to a decline in sales for high-carbon firms. High-carbon firms face challenges in responding to policy pressures to reduce emissions, as they struggle to find incentives to adjust production in order to comply with regulatory requirements. This difficulty is compounded by the potential negative effects of reduced production and increased environmental costs on production efficiency and the bargaining power of these firms.

**Table 6:** Heterogeneity of heterogeneity of enterprise life cycles.

*variables*	Growth period	Maturity period	Decline period
	(1)	(2)	(3)
	*mkp*	*mkp*	*mkp*
*did*	0.0484	0.0801***	-0.1308***
	(0.0762)	(0.0161)	(0.0058)
*CV*	*Yes*	*Yes*	*Yes*
*Enterprise FE*	*Yes*	*Yes*	*Yes*
*Time FE*	*Yes*	*Yes*	*Yes*
*Obs*.	1697	1689	1894
*R* ^*2*^	0.6783	0.5236	0.4892

Note: *, **, *** denote significance levels of 10%, 5%, and 1% respectively, and values in parentheses denote robust standard errors.

### 4.3 Incomplete cost pass-through mechanism test

Based on theoretical analysis, the primary factor contributing to the constraint of policy on product bargaining power is the incomplete transmission of cost shocks to product prices, thereby hindering the realization of the innovation compensation effect. To examine this incomplete cost transmission effect, the proxy variable for product bargaining power, the enterprise price markup rate, was disaggregated into product unit price and product marginal cost using the construction formula, as suggested by Lu and Yu [50]. Finally, the impact of the Carbon emissions trading policy on product price and product marginal cost, respectively, was obtained by regression. The variables are constructed as follows.

Product unit price (PP), The product unit price of exporters is calculated based on customs data. Product marginal cost (PC), The marginal cost of the product is derived from the calculation of the enterprise’s price plus rate.

The findings presented in columns (1) and (2) of Table 7 demonstrate significant effects of the carbon trading policy on firm behavior. Specifically, the coefficient of product price (-0.0674) is statistically significant at the 10% level, while the coefficient of marginal product cost (0.0721) is statistically significant at the 1% level. These results suggest that the carbon trading policy leads to a reduction in product prices and an increase in marginal production costs for firms. Consequently, there is evidence supporting the hypothesis that the degree of imperfect cost-price transmission is exacerbated by the implementation of the carbon trading policy.

**Table 7:** Incomplete cost pass-through mechanism test.

*variables*	(1)	(2)
	*PP*	*PC*
*did*	0.0674*	0.0721***
	(0.0383)	(0.0130)
CV	*Yes*	*Yes*
*Enterprise FE*	*Yes*	*Yes*
*Time FE*	*Yes*	*Yes*
*Obs*.	5280	5280
*R* ^*2*^	0.6352	0.6867

Note: *, **, *** denote significance levels of 10%, 5%, and 1% respectively, and values in parentheses denote robust standard errors.

### 4.4 Product quality bottleneck effect of CETP on PBP test

The theoretical foundation of the product quality bottleneck effect was outlined earlier. In order to examine Hypothesis 3, a nonlinear moderated effects model was employed to assess the bottleneck effect of product quality, and product quality indicators were developed.

Product quality (*qua*), referring to the study of Khandelwal et al. [51], the demand information regression backcasting method is used to measure product quality. In contrast to the conventional approach of assessing product quality solely through pricing, this method incorporates additional variables influencing consumer demand, such as export volume. The requisite data for this method are readily accessible, enabling estimation of export product quality through the demand equation. Consequently, this approach has gained significant traction in scholarly investigations. The findings in Column (1) of Table 8 illustrate the outcomes of the nonlinear moderated effects model regression analysis. The results indicate a U-shaped relationship in which the quality of products from high-carbon firms is initially suppressed and subsequently enhanced by the impact of carbon emissions trading policy on the bargaining power of said firms. This indicates that the rise in costs resulting from subpar product quality during the improvement process will further compound the policy’s constraining impact on the market influence of high-carbon enterprises. Conversely, once product quality surpasses the bottleneck, enhancements in quality will yield greater premiums, thereby mitigating the policy’s constraining effect on the market influence of high-carbon enterprises. This study utilizes the China Customs Database to assess the quality of enterprise products, acknowledging that the desensitization of data in the database from 2017 onwards has hindered its compatibility with enterprise databases. As a result, the study focuses on data from the China Customs Database for the years 2010–2016 to evaluate the quality of high-carbon enterprise products. Additionally, the study analyzes the average level of product quality in China’s high carbon industry in 2020 at the national industry level, which serves as a basis for subsequent sections of the study.

**Table 8:** Testing product quality bottleneck effect of carbon trading policies on high-carbon enterprises’ product bargaining power.

*variables*	Nonlinear moderating effect	Subgroup regression	
	(1)	(2)	(3)
	*mkp*	*mkp*	*mkp*
*did*	-0.0406*		
	(0.0207)		
did_*_ *qua*	-0.4480*		
	(0.2191)		
did_*_ *qua* ^*2*^	0.3761**		
	(0.1829)		
did_*_ *quall*		-0.1230***	
		(-0.0082)	
did_*_ *quahl*			0.1088***
			(0.0064)
CV	*Yes*	*Yes*	*Yes*
*Enterprise FE*	*Yes*	*Yes*	*Yes*
*Time FE*	*Yes*	*Yes*	*Yes*
*Obs*.	5280	5280	5280
*R* ^*2*^	0.5446	0.4854	0.6223

Note: *, **, *** denote significance levels of 10%, 5%, and 1% respectively, and values in parentheses denote robust standard errors.

After that, based on the test results in column (1), and according to the formula $X=-\beta_{3}/2\beta_{5}$ to calculate the symmetry axis of model (3), that is, the product quality in the carbon emissions trading pilot policy on the high carbon bargaining power of the product of the moderating role of the "U" inflection point value is the value of the bottleneck. The determined bottleneck value for product quality, denoted as I = 0.5956, surpasses the overall average value of product quality for high-carbon firms in China in 2020, which stands at 0.4268. This suggests that the majority of high-carbon enterprises in China have not yet achieved product quality levels that exceed the bottleneck value [52]. After that, according to the bottleneck value construct the group of dummy variables *quall* and *quahl*, where *quall* represents the low product quality group dummy variable, that is, when the enterprise’s product quality level is lower than the bottleneck value of *I* is set to 1, or else is set to 0; *quahl* represents the high product quality group dummy variable, that is, when the product quality level of the high-carbon enterprises is higher than or equal to the bottleneck value of *I* is set to 1, *quahl* is a high product quality group dummy variable, i.e., set to 1 when the product quality level of high-carbon enterprises is higher than or equal to the bottleneck value *I*, otherwise set to 0 [53]. Next, the group of dummy variables is multiplied by the policy dummy variables in order to examine the influence of carbon emissions trading policy on the bargaining power of high-carbon enterprise products, considering product quality before and after reaching the bottleneck value [54]. The regression findings are displayed in columns (2)-(3) of Table 8, indicating a non-linear impact of carbon emissions trading policy on the bargaining power of high-carbon enterprise products [55]. When the quality of products breaks through the bottleneck value, the carbon emissions trading policy can promote the bargaining power of products of high-carbon enterprises, which verifies Hypothesis 3.

## 5. Conclusions, policy recommendations and prospects

### 5.1 Conclusion

This study investigates the impact of the carbon emissions trading policy on the product bargaining power of emission-control firms in China’s carbon emissions trading market. Utilizing the entry of high-carbon firms into the list of emission-control firms as a quasi-natural experiment, we derive three key conclusions through theoretical analysis and empirical testing: (1) The carbon emissions trading policy significantly reduces the product bargaining power of high-carbon firms. These findings are supported by various robustness tests. The influence of carbon trading policies on high-carbon firms is contingent upon the life cycle characteristics of the firms. Specifically, these policies have been found to enhance the product bargaining power of mature firms, diminish the product bargaining power of declining firms, and have no discernible impact on the product bargaining power of firms in the growth stage. (2) Mechanism test finds that the incomplete transmission effect of cost shocks resulting from carbon emissions trading policies has negatively affect the product bargaining power of high-carbon enterprises. (3) Additional research indicates that the carbon emissions trading policy hinders the product bargaining power of high-carbon firms due to the incomplete transmission effect of environmental cost shocks. Nevertheless, when the product quality surpasses the threshold value of 0.5956, the degree of incomplete cost pass-through is enhanced, resulting in a notable enhancement of the product bargaining power of high-carbon enterprises through the implementation of the policy. Simultaneously, it also indicates that the product quality of the majority of high-carbon enterprises in China has not reached the inflection point, making it challenging to leverage quality to enhance pricing power.

### 5.2 Policy recommendations

The policy implications of this study are as follows:

First, the establishment of a low-carbon industrial development fund to stabilize the capital chain of enterprise R&D investment. The government should set up an industrial development fund for the introduction of green technology and the renewal of environmental protection equipment and increase the investment and subsidies for the upgrading of pollution control technology and the renewal of energy-saving and emission reduction equipment of high-carbon enterprises. The implementation of a carbon emissions trading mechanism can help offset the short-term cost escalation faced by high-carbon enterprises as a result of environmental regulations, mitigate the adverse effects of environmental compliance expenses on the research and development (R&D) endeavors of high-carbon enterprises, and enhance the consistency and efficacy of R&D investments by high-carbon enterprises as they transition towards becoming high-end enterprises.

Furthermore, it is recommended to enhance policy support for high-carbon enterprises in improving product quality by utilizing the carbon emissions trading market platform. The government should consider issuing green qualification certificates to eligible high-carbon enterprises in order to facilitate their access to "green financing". Additionally, it is imperative for this policy to align cohesively with other incentive measures, bolster investment in technological research and development for firms facing challenges in quality enhancement, facilitate the marketization and industrialization of technological innovation achievements, and incentivize high-carbon enterprises to integrate product quality considerations into their innovation-driven development strategies.

Thirdly, it is recommended that differentiated carbon quota constraints be implemented based on the industry characteristics and product features of various high-carbon enterprises. The government should prioritize meticulous and progressive policymaking, enhance the initial allocation, accounting, and other mechanisms of the carbon market, and establish a more rational guide price for carbon emission rights in high-carbon industries. These measures aim to incentivize high-carbon enterprises to enhance their product quality and upgrade their operations. Simultaneously, it is recommended to decrease the inclusion criteria for emission-control enterprises in the carbon market, thereby expanding the participation of such enterprises in carbon emissions trading. This approach aims to establish incentives and constraints for high-carbon enterprises to reduce emissions effectively.

### 5.3 Limitations and prospects

While this paper thoroughly examines the inherent relationship and impact mechanism between carbon emissions trading policy and product bargaining power, several gaps remain. Specifically, the analysis primarily focuses on the incomplete transmission effect of costs as the factor inhibiting the product bargaining power of high-carbon enterprises. Future research could delve into additional micro-influencing mechanisms. Furthermore, the scope of heterogeneity analysis in this study is constrained by the available data, suggesting that future research could benefit from acquiring more comprehensive data to facilitate a more in-depth exploration of heterogeneity.

## Supporting information

S1 File (DOCX)

## Abbreviations

Simplified Form: Full Name
CETP: carbon emissions trading policies
PBP: product bargaining power
H-C: high-carbon
