# Supplementary material for: Can carbon emissions trading policy promote product bargaining power increases for high-carbon enterprises? Evidence from China
Source: PLoS One. 2024 Jun 17;19(6):e0302916. doi: 10.1371/journal.pone.0302916 (PMC11182541; doi:10.1371/journal.pone.0302916)
Supplement: Supplementary file 1 [file pone.0302916.s001.docx]

some of raw data
used in study.xlsx
